# Genetic characterization of Polish ccRCC patients: somatic mutation analysis of *PBRM1*, *BAP1* and *KDMC5*, genomic SNP array analysis in tumor biopsy and preliminary results of chromosome aberrations analysis in plasma cell free DNA

**DOI:** 10.18632/oncotarget.15331

**Published:** 2017-02-15

**Authors:** Katarzyna Kluzek, Malgorzata I. Srebniak, Weronika Majer, Agnieszka Ida, Tomasz Milecki, Kinga Huminska, Robert M. van der Helm, Adrian Silesian, Tomasz M. Wrzesinski, Jacek Wojciechowicz, Berna H. Beverloo, Zbigniew Kwias, Hans A.R. Bluyssen, Joanna Wesoly

**Affiliations:** ^1^ Department of Human Molecular Genetics, Institute of Molecular Biology and Biotechnology, Adam Mickiewicz University in Poznan, 61-614 Poznan, Poland; ^2^ Department of Clinical Genetics, Erasmus Medical Center, 3015 CN Rotterdam, The Netherlands; ^3^ Laboratory of High Throughput Technologies, Institute of Molecular Biology and Biotechnology, Adam Mickiewicz University in Poznan, 61-614 Poznan, Poland; ^4^ Department of Urology and Urological Oncology, Poznan University of Medical Sciences, 61-285 Poznan, Poland; ^5^ Genomic Laboratory, DNA Research Center, 61-612 Poznan, Poland

**Keywords:** ccRCC, SNP microarrays, SNV, chromatin remodeling, cell free DNA

## Abstract

**Background:**

Mutation analysis and cytogenetic testing in clear cell renal cell carcinoma (ccRCC) is not yet implemented in a routine diagnostics of ccRCC.

**Material and methods:**

We characterized the chromosomal alterations in 83 ccRCC tumors from Polish patients using whole genome SNP genotyping assay. Moreover, the utility of next generation sequencing of cell free DNA (cfDNA) in patients plasma as a potential tool for non-invasive cytogenetic analysis was tested. Additionally, tumor specific somatic mutations in *PBRM1*, *BAP1* and *KDM5C* were determined

**Results:**

We confirmed a correlation between deletions at 9p and higher tumor size, and deletion of chromosome 20 and the survival time. In Fuhrman grade 1, only aberrations of 3p and 8p deletion, gain of 5q and 13q and gains of chromosome 7 and 16 were present. The number of aberrations increased with Fuhrman grade, all chromosomes displayed cytogenetic changes in G3 and G4. ccRCC specific chromosome aberrations were observed in cfDNA, although discrepancies were found between cfDNA and tumor samples. In total 12 common and 94 rare variants were detected in *PBRM1*, *BAP1* and *KDM5C*, with four potentially pathogenic variants. We observed markedly lower mutation load in *PBRM1*.

**Conclusions:**

Cytogenetic analysis of cfDNA may allow more accurate diagnosis of tumor aberrations and therefore the correlation between the chromosome aberrations in cfDNA and clinical outcome should be studied in larger cohorts. The functional studies on in *BAP1*, *KDM5C*, *PBRM1* mutations in large, independent sample set would be necessary for the assessment of their prognostic and diagnostic potential.

## INTRODUCTION

Kidney cancer represents 2–3% of all adult malign-ancies diagnosed annually [1]. Renal cell carcinomas (RCC) originating from the renal cortex are the most common form of all primary renal neoplasms. RCC consists of several histological subtypes with distinct molecular alterations and clinical outcomes, the most common (70-80% of cases) and aggressive being clear cell renal cell carcinoma (ccRCC) [2].

In over 70% of sporadic ccRCC cases, von Hippel–Lindau (*VHL*) tumor suppressor gene inactivation through sequence alteration, promoter CpG hypermethylation or loss of heterozygosity has been reported [3–5]. Germline *VHL* mutations are also linked to an increased risk of developing ccRCC in patients with the inherited disorder von Hippel–Lindau syndrome [6]. VHL protein (pVHL) is involved in many cellular processes. Its best characterized function is the regulation of response to oxygen level changes by targeting hypoxia-inducible factors (HIFs) for ubiquitin-dependent proteasomal degradation [6]. pVHL inactivation results in constitutive HIFs activity leading to enhanced angiogenesis and cell proliferation thus stimulating tumor growth. Recent studies showed that in cell lines and mice defective in pVHL mitotic spindle checkpoint function is impaired, contributing to chromosomal instability that may stimulate tumor progression [7, 8].

*VHL* mutations are not the only driving force in ccRCC tumorigenesis. Recent large-scale sequencing studies have revealed frequent mutations affecting chromatin modifying genes such as *PBRM1*, *BAP1*, *SETD2*, *KDM5C* and *KMD6A* [9–11]. *PBRM1*, *SETD2* and *BAP1*, similarly to *VHL*, map to the 3p21 and this suggests that essential component of ccRCC pathogenesis may be allelic loss of 3p resulting in haploinsufficiency for four tumor suppressors simultaneously. Moreover, mutations in *BAP1* and *SETD2* are probably secondary events to *VHL* or *PBRM1* loss [12, 13]. Sequencing projects have also identified potentially pathogenic mutations of the known tumor suppressor genes *CDKN2A*, *TP53*, *NF2*, and *PTEN* in a subset of ccRCC tumors [9] as well as mutations in PI3K pathway regulators *mTOR* and *PIK3CA*, frequently abolished in many cancers [14].

In addition to gene mutations, chromosomal alterations characteristic for ccRCC have been identified in a number of high-resolution cytogenetic tumor studies on a genome-wide level, including array-based comparative genomic hybridization (aCGH) and high-density SNP genotyping arrays [15–18]. The most frequent genomic changes are deletion of chromosome 3p harboring *VHL* gene (70-80%) and/or loss of heterozygosity (LOH) at 3p, as well as gain of chromosome 5q (50–60%) [5]. Other chromosomal imbalances have also been reported, in a large study encompassing 763 patients with ccRCC from Central and Eastern European population the most common were deletion of chromosome 14q (46.8%), 8p (38.1%), 4q (35.4%), 9p (32.3%), 6q (30.8%), 1p (23.5%) and gains of 7q (39.6%), 7p (30.6%), 20q (25.5%), 12q (24.8%) and 12p (22.8%) [18].

Exploring the molecular alterations, beyond better characteristics of pathology and etiology of ccRCC, may also have potential clinical implications. *VHL* inactivation may serve an important diagnostic factor, however its prognostic relevance in patients with sporadic ccRCCs remains undetermined due to low number of studies performed and the conflicting results [19–21]. On the other hand, most of mutations of chromatin modulating genes are associated with advanced stage, metastases, and shorter overall survival [12, 22]. Copy number alterations in ccRCC tumors have also shown association with clinical parameters. Interestingly, deletion of 3p (accompanied by other chromosomal aberrations) is correlated with improved survival, low tumor stage and grade, and low risk of distant metastases [16, 23]. 4p, 9p and 14q deletions and 7q, 8q, 20q gains are correlated with higher stage, grade, and/or worse prognosis [16, 24–27]. Additionally, 1q, 7q, 12q and 20q gains and deletions of 9p have been associated with metastatic risk [27]. Moreover, aCGH profiling of ccRCC tumors performed by Moore *et al*. revealed that regardless of stage and grade, more aberrations were observed in tumors derived from male patients than from females [18].

Despite of the growing understanding of the mechanisms leading to ccRCC, the molecular pathogenesis of the disease remains rather poorly understood and molecular testing of cytogenetic alterations is not yet implemented in a routine diagnostics of ccRCC [28]. To widen our knowledge on genetic events involved in tumor initiation and progression, we performed whole-genome SNP genotyping in 83 ccRCC tumors from Polish population. In addition, in proof of concept experiment, we also attempted to detect cancer-specific chromosome aberrations in circulating cell-free tumor-derived DNA (cfDNA) since this non-invasive approach has a great potential in developing new molecular or cytogenetic methods for cancer diagnosis and prognosis [29, 30]. The analysis of the ccRCC tumors was extended with the sequencing of three genes frequently mutated in ccRCC: *PBRM1*, *BAP1* and *KDM5C* in order to investigate the extent of the alterations in those genes in Polish population of ccRCC patients.

## RESULTS

### Chromosomal analysis in ccRCC tumors

A total of 83 ccRCC and 12 normal tissues were screened by genomic SNP array analysis. An aberrant array profile was observed in 73 ccRCCs; 10 tumors showed no genomic changes. Five tetraploid or almost tetraploid samples showing extremely large number of secondary aberrations were excluded from the study as the imbalances highly influenced the meta-analysis of our study. Further analysis was performed on 78 tissues: 68 chromosomally abnormal and 10 chromosomally normal samples. No acquired genomic copy number variants (CNVs) >0.5Mb were detected in the 12 control, randomly selected, paired kidney tissues samples collected from areas histopathological identified as non-tumor tissue.

**Figure 1:**
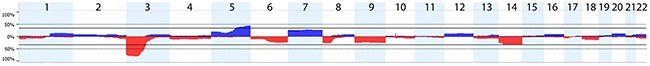
Graphical representation of the most common ccRCC chromosomal imbalances detected by high-resolution SNP array analysis The boxes indicate individual chromosomes with p- and q-arm, respectively. Blue bars signify gains, red bars - deletions. Only the somatic chromosomes are depicted on the graph.

We identified 28 genomic segments with common changes, defined as occurring in ≥5% of the ccRCC tumors (Table 1). The general pattern and frequency of somatic alterations in the analyzed cohort of tumor samples is shown in Figure 1. Significant loss of genetic material was found on chromosomes 3p (n=64, 82%), 14q (n=28, 36%), 4/4q (n=21, 27%), 8 (n=21, 27%), and 9/9p (n=20, 26%). The most significant gain of genetic material was detected on chromosome 5q (n=30, 39%) and chromosome 7 (n=21, 27%). In 24 samples ≥10 CNVs were observed. This group is characterized by downregulation of *VHL* (n=18), as well as high tumor stage (T3/4, n=13) and grade (G3/4, n=20) suggesting rather advanced disease. In one case we detected as many as 39 CNVs, clearly associated with a very severe phenotype: the patient presented with high tumor stage, grade 4, lung metastases, died within 24 months after nephrectomy.

**Table 1:** Frequency of chromosomal aberrations in patients with ccRCC (n=78). Only aberrations present in ≥5% of samples are shown

Type of aberration	Frequency
**3p deletion (including *VHL***)	82.1% (64)
**5q gain (including *STC2***)	43.6% (34)
**5q gain (including *CSF1R***)	38.5% (30)
**Monosomy 14**	35.9% (28)
**4q deletion (including *NEIL*) or monosomy 4**	26.9% (21)
**7q gain (including *NAMPT* and *MCM7*) or trisomy 7**	26.9% (21)
**8p deletion (including *DLC1* and *NRG1***)	26.9% (21)
**9p deletion (including *CDKN2A*) or monosomy 9**	25.6% (20)
**Chromothripsis (>4 breakpoint on one chromosome arm)**	24.4% (19)
**6q deletion (including *PARK2*) or monosomy 6**	24.4% (19)
**8p deletion (including *DLC1*, *NRG1* and *SFRP1***)	21.8% (17)
**Y chromosome deletion (in 17 out of 45 male patients)**	21.8% (17)
**5p gain (5p15)**	17.9% (14)
**5q gain (including *VCAN***)	15.4% (12)
**18q deletion (including *DCC***)	15.4% (12)
**1q gain**	12.8% (10)
**1p deletion (including *RUNX3 and ARID1A***)	11.5% (9)
**12q gain or trisomy 12 (including *CDK4* and *NDUFA4L2***)	10.3% (9)
**13q deletion (including *RB1***)	10.3% (8)
**20q gain (including *E2F1* and *HCK*) or trisomy 20**	10.3% (8)
**Monosomy 22**	10.3% (8)
**2q gain (including *ZNF804A***)	7.7% (6)
**8q gain (including *COL14A1***)	7.7% (6)
**10q deletion (including *KLLN***)	7.7% (6)
**17p deletion (including *BP53***)	7.7% (6)
**2q deletion**	6.4% (5)
**3q gain**	6.4% (5)
**16q gain (including *CDH1***)	6.4% (5)
**13q gain (including *EDNRB***)	5.1% (4)

Numbers in parentheses indicate number of patients.

### Association of the chromosomal aberrations to metastatic stage, TNM stage and survival

Tumor stage assessed by TNM scale is an important factor in determining the tumor status. We observed differences in the distribution of 28 most common genomic changes between high pT stage groups 3 and 4 (larger size of the primary tumor) and low pT stage groups (1 and 2), however only deletion of the 9p reached the level of significance (p=0.038). Univariate logistic regression showed highest odds ratio for pT stage 3, 4 (OR 3.5, 95% CI OR 1.2-11.6, p=0.03, Table 2) in case of this alteration, suggesting that presence of 9p deletion could be used as potential predictor of pT parameters. We also grouped ccRCC tumors according to the presence of metastases and the regional lymph nodes involvement (N) and subsequently analyzed their cytogenetic alterations, but no statistically significant correlation between the parameters were found in our cohort (data not shown).

**Table 2:** Univariate logistic regression analysis of association between high pT (3, 4), high Fuhrman grade (3, 4) and chromosomal changes in ccRCC tumors

	CNV	OR	95% CI	P-value
**pT**	***9p Del***	3.5	1.2-11.6	0.03
**Fuhrman grade**	***Monosomy 14***	2.9	1.06-8.54	0.04
	***18q Del***	11.4	2.01-215.7	0.02
	***9p Del***	4.5	1.42-17.74	0.01

**Figure 2:**
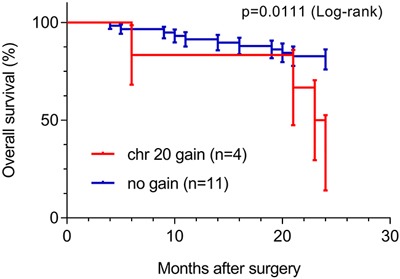
Overall survival analysis of sporadic ccRCC patients (n=64, 24 month follow-up), based on chromosome 20 copy number alteration Study included 6 patients with chr20 gain and 58 patients with normal CN.

Moreover, we investigated association of all chromosomal aberrations presented in Table 1 with 24 months survival. For 75 patients clinical follow up was available, six patients were excluded from survival analysis: one patient died one month after diagnosis due to surgery complications and the follow-up of 5 patients was shorter than 2 years. Statistical analysis (Fisher's exact test, n=69) showed significantly shorter survival for patients with tumors containing gain of chromosome 20 (p=0.0286) compared with cases without this aberration. This observation was further supported by Kaplan-Meier survival analysis with log-rank test p=0.011, as presented at Figure 2. We next evaluated the independent prognostic value of chromosome 20 gain using Cox proportional hazards model. Regression analysis showed that chromosome 20 gain was significantly associated with survival rate, suggesting that its presence can be used as a prognostic factor for increased risk of death (HR 4.3; 95%CI 1.3-13.99; p=0.015), as suggested previously [27].

### Chromosomal aberrations involved in the transition to higher Fuhrman grade

**Figure 3:**
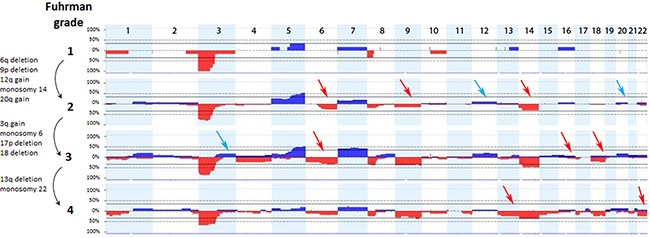
Chromosomal imbalances associated with grading of ccRCC tumors The analysis was performed on 77 tumors: grade 1 (n=6), grade 2 (n=26), grade 3 (n=29), grade 4 (n=16). Arrows indicate successive acquisition of chromosomal abnormalities (gains – blue, deletions - red) with increasing Fuhrman grade, depicted on the left side of the graph.

We analyzed cytogenetic alterations in ccRCC tumors according to Fuhrman tumor grade. Tumors at the lowest grade, G1, had typically only a few cytogenetic alterations: deletion of 3p, 1p, 8p, 10q and gain of 5q, 7, 13q and 16q (Figure 3, Supplementary Table 1). The number of large aberrations increased with Fuhrman grade: in G2 only chromosomes 15, 17, 19 and 21 showed no alterations while tumors at the latest grades, G3 and G4, displayed cytogenetic changes affecting all chromosomes. Our data support the notion that chromosomal changes may be gradually acquired during tumor progression from low to high Fuhrman grade. At this stage none of these observations reached statistical significance in Fisher's exact test, due to small sample size (data not shown, see discussion).

We observed that deletions of 9p (p=0.015) and 18q (p=0.034) were more common in tumors with high Fuhrman grade (3, 4). Value of these chromosomal alterations as potential predictors of Fuhrman tumor grade (as well as monosomy 14) was confirmed by univariate logistic regression which showed highest odds ratio to higher grades (G3, G4) in case of these three alterations (OR 4.5, 95%CI 1.42-17.74; OR 11.4, 95%CI, 2.01-215.7; OR 2.9, 95%CI, 1.06-8.54, respectively; Table 2).

### Analysis of ccRCC tumor derived chromosomal aberrations in circulating cell free DNA

**Figure 4:**
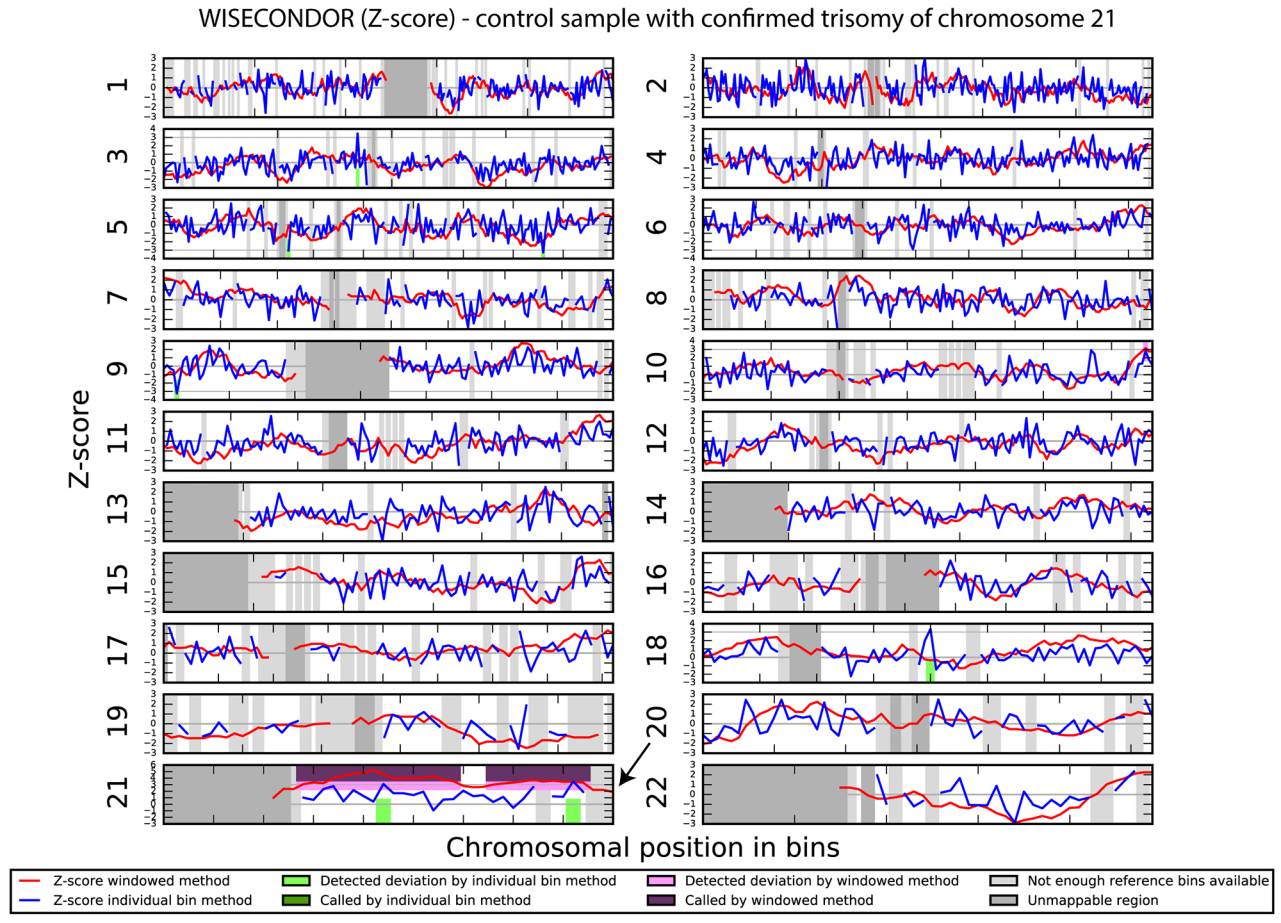
Chromosomal aberrations in cfDNA detected by shallow sequencing using WISECONDOR software Aberration calling results from the sliding window method and the individual bin method. Positive control shows trisomy 21 in cfDNA.

**Figure 5:**
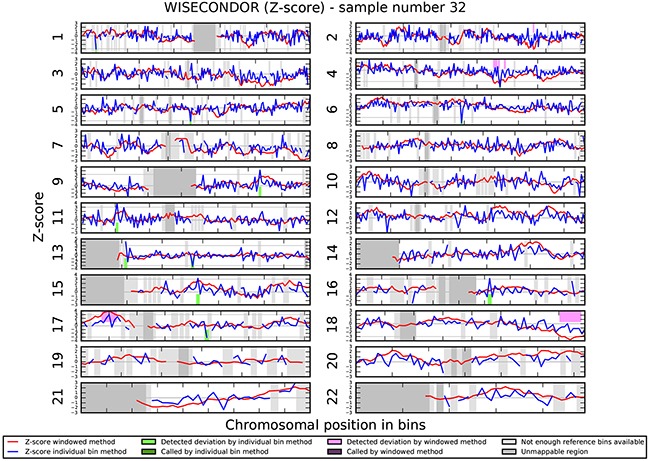
Chromosomal aberrations in cfDNA Negative control (sample 32) shows no chromosomal aberrations.

**Figure 6:**
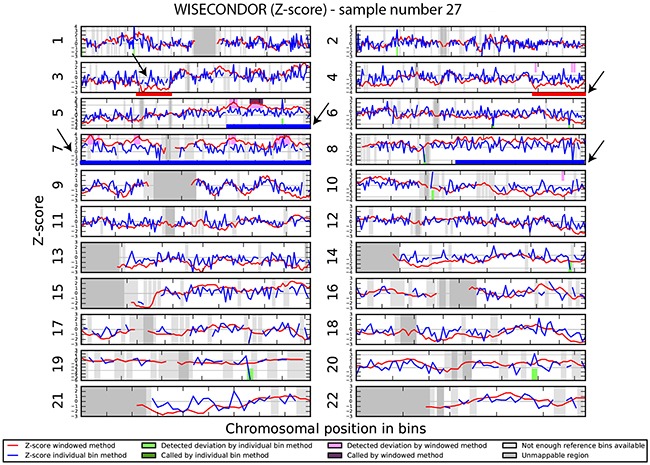
Chromosomal aberrations in cfDNA Sample 27, selected as negative control, shows very low mosaic chromosome aberrations: 3p deletion (red line), 5q gain (blue line) as well as mosaic trisomy 7 and possibly 8q gain and 4q deletion.

**Figure 7:**
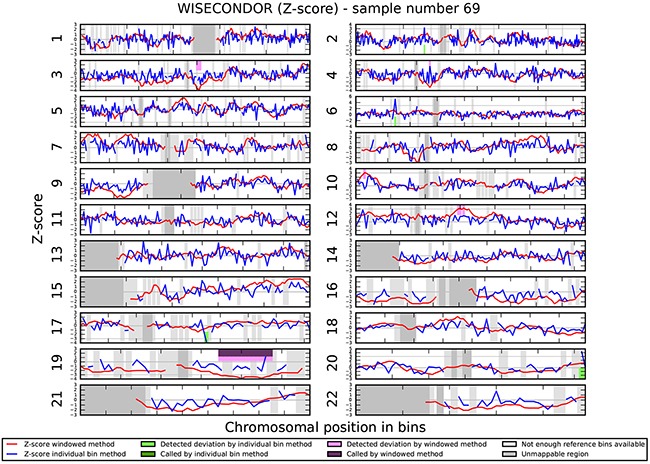
Chromosomal aberrations in cfDNA. No chromosomal aberrations were detected in sample 69

**Figure 8:**
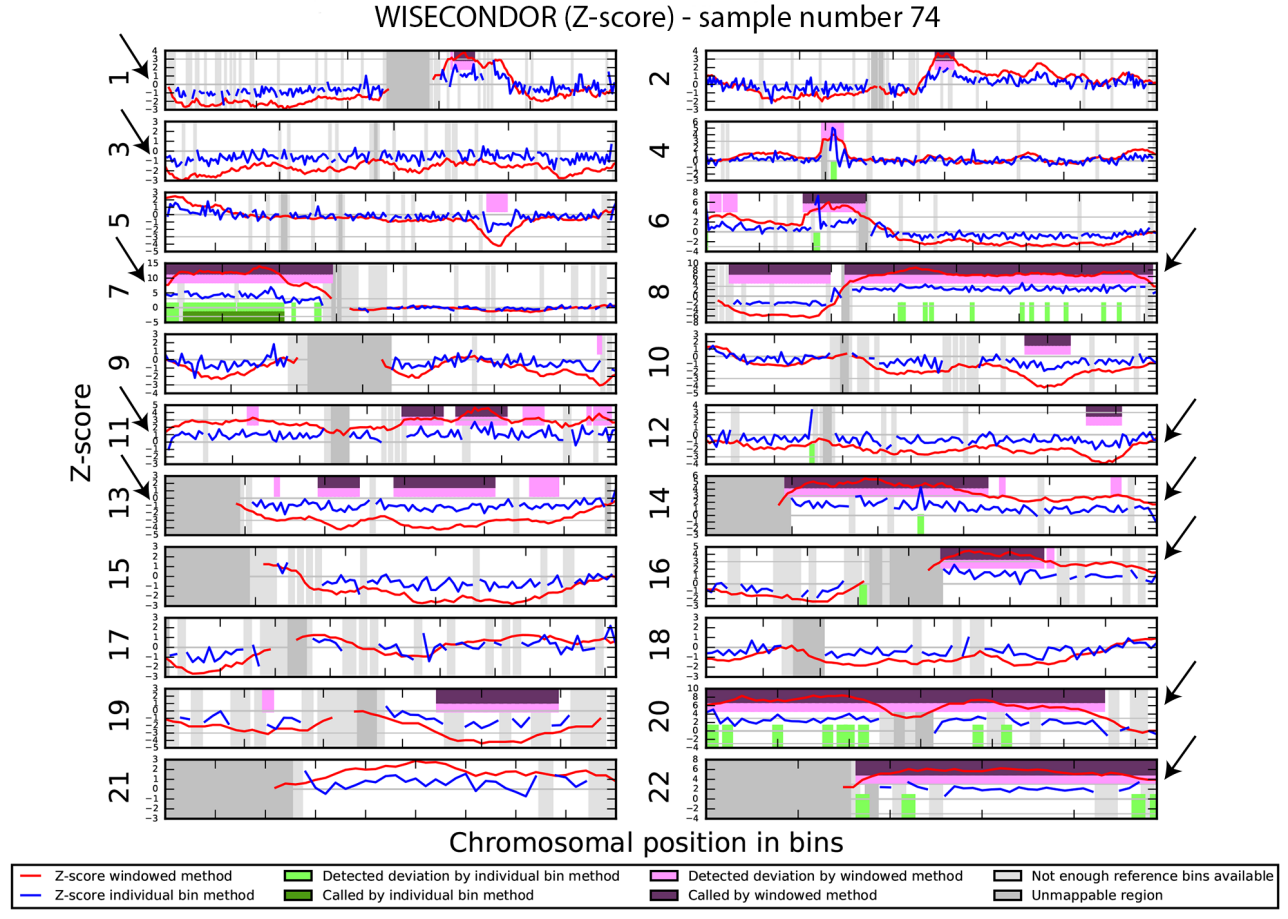
Chromosomal aberrations in cfDNA Sample 74 shows clear multiple chromosome aberrations characteristic for ccRCC: a.o. deletion of chromosome 1, deletion of chromosome 3, 7p gain, 8p-, 8q gain, trisomy 11, deletion of chromosome 13, trisomy 14, 16q gain, trisomy 20 and trisomy 22.

We investigated if the aberrations present in the tumor could be detected in plasma-derived circulating cell free DNA (cfDNA). In this proof of concept experiment two patients for which SNP array analysis did not detect chromosomal aberrations and two patients with maximum 2 large chromosomal alterations (>75 Mb) were selected for cfDNA testing in plasma. cfDNA was isolated from 3ml of plasma and the concentration of extracted cfDNA ranged from 0.5 - 15.5 ng/μl. Prior sequencing, quality of prepared libraries was checked on Agilent Bionalyzer (data not shown), with an average library size of 300 bp. During SR-50 sequencing experiment ~23mln mapped reads per sample were obtained on average, with genomic coverage ranging from 0.17 to 0.57. A sample with trisomy 21 was used as a positive control and was correctly identified as trisomy 21, proving the accuracy of the assay (Figure 4). ccRCC specific chromosome aberrations were detected, but discrepancies between tumor microarray and plasma cfDNA from patients’ plasma results were observed as summarized in Table 3. Sample 32 showed a normal cfDNA result (Figure 5), which was in line with microarray results. Sample 27 showed no chromosomal aberrations on microarray analysis of the tumor biopsy, however ccRCC specific aberrations could be noted on WISECONDOR plot. Very subtle aberrations were present in this sample: interstitial 3p deletion, 5q gain and low mosaic trisomy 7 and possibly 4q deletion and 8q gain (Figure 6). Sample 69 showed no chromosomal aberrations in cfDNA analysis (Figure 7), although microarray on tumor biopsy revealed 3p deletion and 5q gain. Strikingly sample 74 very clearly showed multiple aberrations in cfDNA analysis, which are characteristic for ccRCC (Figure 8), such as mosaic loss of the entire chromosome 3, whereas microarray analysis showed 3p deletion only.

**Table 3:** Comparison of SNP array analysis of tumor samples and cfDNA derived from plasma of ccRCC patients

Sample number	T	N	M	F	SNP array analysis	cfDNA sequencing*	cfDNA mapped reads	Conclusions
**32**	pT1a	0	no	G2	normal results	normal results	26.8 mln	cfDNA results are fully in line with microarray analysis of tumor sample.
**27**	pT3a	0	no	G4	normal results	very low mosaic aberrations: 3p deletion, 4q deletion, 5q gain, chr7 gain, 8q gain	15.8 mln	cfDNA probably more representative than a single biopsy, normal array results are likely due to tumor heterogeneity. In this case cfDNA could have potentially prognostic value.
**69**	pT1a	0	yes	G3	abnormal results: 3p deletion, 3q deletion, 5q gain	normal results	40.8 mln	Discrepant result can be explained possibly by low primary tumor cfDNA fraction, which can cause false negative results. This patient should be resampled, however it was not possible in our research settings.
**74**	pT4	NA	no	NA	abnormal results: 3p deletion	evident complex chromosome aberrations: 1p deletion, chr3 deletion, 7p gain, 8p deletion, 8q gain, chr11 gain, chr12 deletion, chr13 deletion, chr14 gain, 16q gain, chr20 gain, chr22 gain	19.1 mln	cfDNA showed multiple aberrations typical for ccRCC, whereas the biopsy showed only 3p deletion. cfDNA is probably more representative than a single biopsy, due to heterogeneity of the tumor. In this case cfDNA could have potentially prognostic value as chr20 gain was visualized by cfDNA and not by microarray on a single biopsy.

* Aberrations smaller than 20Mb and concerning chromosome 19 are not listed due to the limitations of the method

Abbreviations: T, tumor size; N, lymph node involvement; M, metastasis; F, Fuhrman grade; NA, data not available

### Chromosomal aberrations in *PBRM1*, *BAP1* and *KDM5C loci*

The first comprehensive analysis of genes mutated in ccRCC was performed in the The Cancer Genome Atlas project [4]. Newly identified genes, involved in chromatin remodeling, were represented by *PBRM1*, *BAP1*, *SETD2*, *KDM5C* and *KMD6A*. Here, in order to specifically identify chromosomal rearrangements spanning *PBRM1*, *BAP1* and *KDM5C* in the cohort of Polish ccRCC patients, we performed fine mapping of these loci with a cut-off 0.5 MB. *PBRM1* and *BAP1* are located at the short arm of chromosome 3 (3p21.1 and 3p21, respectively), in a vicinity of *VHL*; *KDM5C* is located on chromosome X (Xp11.22-p11.21). In the majority of the investigated cases all 3 genes are deleted (n=60). Only in one sample showing *VHL* and *PBMR1* deletion we observed normal copy number of *BAP1*. In one sample we detected normal copy number of *VHL* with *BAP1*, *PBRM1* deletion, however the region 3p25 showed LOH (data not shown). We believe that the analysis of LOH is very relevant in case of individual samples, where chromosome analysis did not show 3p deletion. However, during the analysis on the cohort-level no enrichment in 3p LOH was observed and only one sample mentioned above displayed 3p LOH. *KDM5C* mosaic gain was observed in 2 samples: one showing trisomy X and the second showing structural X chromosome rearrangement. *KDM5C* mosaic deletion was detected in 3 samples, all with mosaic chromosome X monosomy. None of these patients were tested for constitutional karyotyping, and in consequence we cannot exclude the germline origin of these aberrations.

### Identification of mutations in *PBRM1*, *BAP1* and *KDM5C* in Polish ccRCC patients

To further characterize our patient's cohort and identify frequently occurring mutations in ccRCC tumors we performed targeted amplicon sequencing of the entire coding regions and adjacent intronic sequences of the *PBRM1*, *BAP1* and *KDM5C* genes. 28 amplicons with length between 1599 – 5341 nucleotides were generated. The sequencing run yielded a total of 3.86 Gbases of data, with 85% of bases having a Phred quality score (Q score) ≥Q30, with cluster density of 898 ± 27 K/mm2, with 75.21 ± 6.45% of the clusters passing QC filters. Average total number of reads was 56 727 (ranging from1751 to 90216) out of which 88.4% was aligned to target regions. The observed read depth in 82 samples was >20 per base with Q≥30 (average coverage 72.9, range 22.2-122.5). Samples with coverage below 20x were excluded from the analysis.

**Figure 9:**
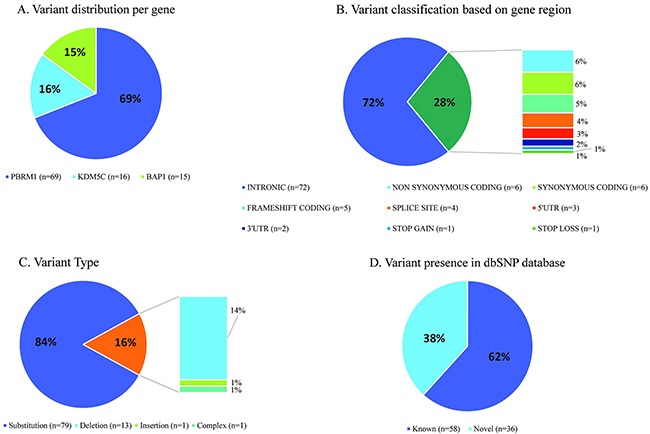
Statistics of somatic variant calling in tumors derived from Polish ccRCC patients

**Figure 10:**
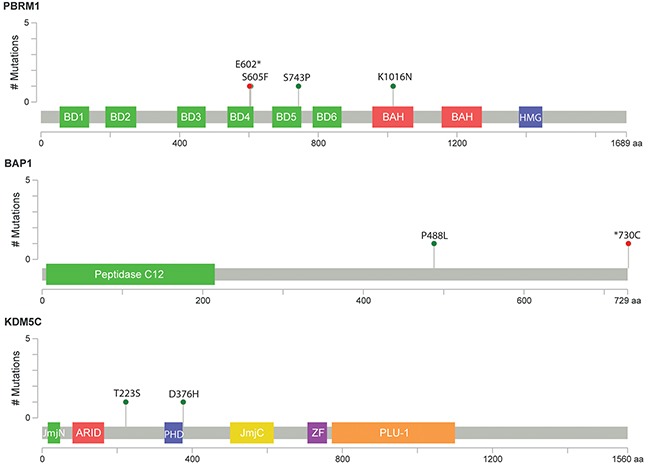
Schematic representation of relative positions of potential mutations within *KDM5C*, *BAP1* and *PBRM1* transcripts Symbols indicate: green circles, missense mutation; red circles, nonsense mutation. BD1-6: bromodomain 1-6; BAH1 and BAH2: bromo-adjacent homology domains 1 and 2; HMG: high mobility group domain; UCH: Peptidase C12: belongs to ubiquitin C-terminal hydrolase family; JmjN and JmjC belong to jumonji family of transcription factors; ARID: AT-rich interaction domain; PH, ZF: zinc finger domains; PLU1: PLU-1-like protein. Figures were generated with cBio MutationMapper [60, 61].

Variant calling performed with StrandNGS resulted in the identification of 12 common and 94 rare variants, present in ≤10% of patients (Supplementary Table 2 and 3, respectively). Common variants were found only in *KDM5C* (n=7) and *PBRM1* (n=5) genes. All except for one were located in intronic sequences, 3 were known, 6 were novel and 3 have been reported as single nucleotide substitutions by dbSNP, but in our sample set we observed small deletions and insertion at the reported positions (for details see Supplementary Table 2). As shown in Figure 9A the majority of rare variants were present in *PBRM1* (69%, n=69), the remaining variants were present at nearly equal frequencies in *KDM5C* (16%, n=16) and *BAP1* (15%, n=15). 70% of all identified somatic variants were intronic and 85% represent substitution type (Figure 9B and 9C). In 10 patients (12%) we found >3 variants in *PBRM1*, with prevalence of intronic mutations. 38% of all identified variants (n=42) were not previously reported, 62% were reported in dbSNP (Figure 9D). In further analysis we focused on variants present in UTR’s, coding sequence and splice sites that can have potential functional impact on encoded protein, and discarded all synonymous and intronic alterations. To determine the functional significance of identified single nucleotide and complex variants we utilized Variant Effect Predictor (Ensembl) and MutationAssessor r3. 23 variants were taken into account, out of which four were predicted to be deleterious and possibly/probably damaging, all present in single samples (Table 4). One novel *KDM5C* variant is located outside of the peptidaseC12 functional domain leading to an amino acid change D376H (Figure 10). The remaining three variants were found in *PBRM1* gene (Figure 10), of which two are novel (p.S743P, p.K1016N). Variant p.S605F was previously described in melanoma and kidney tumor samples (COSMIC database) [31–33]. All possibly/probably damaging *PBRM1* variants were present in functional domains: bromodomain 5, bromo-adjacent homology domain 1 and bromodomain 4, respectively. Due to very limited number of individuals with the mutations we were not able to correlate their presence with the clinical parameters, nor to test their prognostic and diagnostic utility.

**Table 4:** Clinical characteristics of the ccRCC patients, n = 83

Clinical parameter	Number of patients
**Age (years)**	30-87 (median 63.2)
**Males/Females**	45 (54.2%)/38 (45.8%)
**Clinical outcome**	
**Alive (≥24months after nephrectomy)**	62 (79.5%)
**Deceased**	16 (20.5%)
**Follow-up time (months)**	1-55
**pT**	
**1**	39 (47.5%)
**2**	4 (4.9%)
**3**	35 (42.7%)
**4**	4 (4.9%)
**Fuhrman grade**	
**1**	6 (7.4%)
**2**	27 (33.3%)
**3**	30 (37%)
**4**	18 (22.2%)

## DISCUSSION

### Chromosomal aberrations in ccRCC tumor biopsies

SNP microarray analysis was performed to characterize the chromosome aberrations in the cohort of Polish patients with ccRCC. Genome-wide analyses indicated that deletions at chromosomal arms 3p, as well as gains of 5q and 7, are indispensable for the development of ccRCC [18, 26, 34–37] and our data showed that these were the most common chromosome aberrations in our cohort as well. Moreover, deletions at 1p, 4p, 4q, 8p, 9p, 9q, 13q, 14q, and 18q have been found to correlate with higher malignancy grades and tumor stages in ccRCC [15, 18, 25, 26, 36, 38–42]. Our data are in line with these findings, showing a correlation between deletions at 9p and with higher pT. Unfortunately we did not find a correlation between chromosomal rearrangements in metastatic tumors and patient survival, most probably due to relatively short 24 month patient follow-up. We did however detect a statistically significant correlation between deletion of chromosome 20 and the survival time, which was noted before as well, but not as a common finding [27].

As the majority of ccRCC tumors are diagnosed in the advanced metastatic stage resulting in dramatic decrease of patient survival, the number of grade 1 tumors is very low in all publically available published data and very often samples with grade 1 and 2 were analyzed as one group. We were especially interested to investigate early grade samples to search for the primary chromosome aberrations and to identify the ones likely involved in tumor progression. Only 3p and 8p deletion as well as 5q, 7, 13q and 16 gains were seen in the Fuhrman grade 1. To support our findings we analyzed additionally 10 freely accessible samples with grade 1 from ccRCC tumor samples in NEXUS DB (http://www.biodiscovery.com/genomic-databases-nexus-db) (the cohort of publically available data from The Cancer Genome Atlas (TCGA) (http://cancergenome.nih.gov). All samples with grade 1 except one confirmed our results presented in Figure 3. This additional analysis may support the fact that the Fuhrman grade assessed microscopically is less accurate that genome wide SNP analysis. The recognition of primary aberrations and prognostic secondary chromosome aberrations is crucial if non-invasive genetic testing for the diagnosis and follow up of ccRCC tumor would become clinically available.

### Chromosomal aberrations in cell free DNA

Analysis of circulating cfDNA (liquid biopsies) is a promising alternative for classical invasive tissue biopsies and has a great potential in developing new molecular or cytogenetic methods for cancer diagnosis and prognosis [29, 30]. Advances in technology, including allele-specific PCR, digital PCR, MethyLight PCR, next generation sequencing (NGS) and microarrays, allow analyses of circulating cfDNA [43]. However, the accurate detection of copy number alterations is still challenging since only a very small fraction of total cfDNA is derived from tumors in most patients. The presence of tumor cell-free DNA in plasma of patients with ccRCC has been shown before [44, 45], therefore we attempted to detect the chromosome aberrations characteristic for ccRCC in patients’ plasma using shallow NGS and WISECONDOR software [46]. In our proof of concept experiment we show cfDNA contains more advanced chromosome aberrations than detected by the genomic SNP array on tumor sample. Heterogeneity of tumor samples has been described before [47] and it is accepted that a single tumor biopsy sample may not be fully representative for the whole tumor, especially if the tumor size is very large. We also visually detected a number of CNVs in cfDNA that were not “called” by the software, likely due to low concentration of cfDNA present in the plasma of the patient or too low coverage. Therefore, in future if cfDNA is going to be implemented in a diagnostic setting, the assessment of the tumor cfDNA fraction and determination of reliable sample sequencing coverage may be crucial to avoid false negative results. Also a carefully controlled and standardized sample collection and preparation is a precondition for reliable results [48].

Unfortunately our preliminary cfDNA data did not allow us to correlate our results to clinical outcome. More tests are necessary to assess whether the cfDNA analysis reflects the clinical prognosis in more accurate manner than analysis of a single tumor tissue biopsy and whether the tumor heterogeneity could be overcome by cfDNA testing.

### Mutation analysis in *PBRM1*, *BAP1* and *KDM5C*

Data obtained from The Cancer Genome Atlas Research Network revealed essential role of chromatin remodeling genes (e.g. *PBRM1*, *BAP1* and *KDM5C*) in ccRCC tumorigenesis. However, their contribution to cancer development and the functional consequences of the mutations still require further studies. Here, we aimed to test the frequency of previously reported and to identify novel mutations in these 3 genes. In Polish ccRCC patients SNVs frequency (within coding region and splice sites) in our cohort display similar range as mutation frequency reported previously for *BAP1* (6-17%) and *KDM5C* (4–9%) [49–51]. In case of *PBRM1* we observed markedly lower level of variants in Polish population (11%) than reviewed by Piva and colleagues (26-52%) [51]. However, it should be noted that mutations spectra for all above mentioned genes varies greatly between studies i.a. due to different patient groups, sequencing methods and bioinformatic approaches. Moreover, it may be a characteristic feature for our cohort or result from quite stringent filtering of called variants.

**Figure 11:**
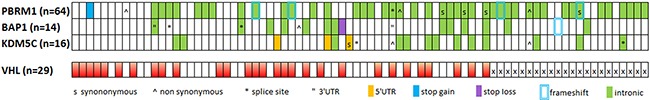
Distribution of variants in affected samples in relation to *VHL* mutations Upper panel shows variant distribution in *PBRM1*, *KDM5C* and *BAP1* genes in 80 ccRCC cases. Matrix represents individual variants in patients samples, color or symbol-coded by type of genetic alteration. Note that intronic variants, often occurring more than once in a single sample, are indicated on the figure as single event. Lower panel shows mutations found in *VHL* gene by Sanger sequencing. All samples with gene alteration are indicated in red, x, data not available.

We did not observe a significant co-occurrence of detected variants and *VHL* mutations (identified with standard Sanger sequencing, data not shown), as demonstrated on Figure 11. Also in contrast to previous reports, variants present in *BAP1* and *PBRM1* were not mutually exclusive in our sample set [13, 33, 50, 51]. *BAP1* loss was reported to correlate with high Fuhrman nuclear grade, higher tumor stage, and worst overall survival [13, 33]. We noted a similar tendency only in case of tumor grade (Supplementary Table 4), but due to the smaller sample size no clear-cut conclusions could be made.

Three novel non-synonymous coding variants and one previously reported were predicted to be damaging and/or deleterious — one in *KDM5C* and three in *PBRM1*, suggesting possible correlation with the ccRCC phenotype. PBRM1 is a member of the SWI/SNF complex involved in chromatin remodeling and contains 6 tandem bromodomains binding lysine residues modified by acetylation [52]. S605F and S743P substitutions are present in bromodomain 4 and 5, respectively, suggesting that they may impair chromatin targeting of PBRM1. Although not present in dbSNP database, p.S605F has been reported in COSMIC in ccRCC patients as well as alterations within residue 743: p.E742_I745del and p.S743F. In addition to bromodomains, two bromo-adjacent homology (BAH) domains, possibly implicated in protein-protein-interactions are present in PBRM1 [53]. These domains are frequent targets of missense mutations in renal cancer, which suggest that K1016N substitution present in BAH1, although not reported before, may have functional impact on the protein function. In contrast, D443H is located outside of KDM5C functional domains, however close to the PHD finger thus we can speculate that this variant could influence rather DNA binding than direct chromatin remodeling functions of this lysine-specific demethylase. Data analysis with Variant Effect Predictor pointed to few more variants as having potentially high functional impact. These include four novel variants leading to frameshift and one introducing premature stop codon within bromodomain 4 at position 602 of *PBRM1*. E602* is not reported in dbSNP/ClinVar but present in COSMIC database and has been found in one Polish patient with ccRCC. The catalytic peptidase C12 domain of BAP1 which has a deubiquitinase activity is often targeted by missense mutations in ccRCC [33]. One variant found in our patients is novel and leads to a frameshift (Figure 10). Loss of STOP codon (*730C) is also novel, but amino acid changes to G or R have been reported previously (COSMIC database) suggesting functional importance of this amino acid change. In conclusion, bioinformatics predictions indicated that 9 out of 19 potentially damaging variants reported in current study may have a functional impact on the proteins and be linked to the ccRCC development. However, it is difficult to predict the consequences of the SNVs in terms of protein function impairment using only computational methods, hence further functional analysis are required to establish their significance. It should be also emphasized that in contrast to *VHL*, in case of *PBRM1*, *BAP1* and *KDM5C* no gene silencing due to promoter hypermethylation has been reported suggesting that gene function impairment is rather entirely achieved through mutations within coding regions and regulatory elements [54]. Therefore, functional studies on in *BAP1*, *KDM5C*, *PBRM1* mutations impact on gene/protein function are required. There is also necessity of population specific analysis of ccRCC mutations (germline and somatic) and their functional consequences, which is supported by our finding that as much as 38% of all identified variants in *BAP1*, *KDMC5*, and *PBRM1* were not previously reported in dbSNP database. This study aimed solely to identify the mutations in the three abovementioned genes in our sample set. Their clinical relevance, in combination with prognostic and diagnostic utility, remain unclear and require additional studies.

## CONCLUSIONS

Our data suggest that whole genome SNP micro-array allows identification of prognostic chromosome aberrations and it may be used in clinical settings especially in borderline case for more accurate Fuhrman grade assessment. However tumor heterogeneity must be taken into account. Chromosome analysis of cfDNA may allow more accurate diagnosis of tumor aberrations and therefore the correlation between the chromosome aberrations in cfDNA and clinical outcome should be studied in larger cohorts. The functional studies on possibly pathogenic variant in *BAP1*, *KDM5C*, and *PBRM1* as well as study of both somatic and germline variations in larger sample sets would be necessary for better risk assessment.

## MATERIALS AND METHODS

### Sample collection

All kidney tissue specimens were collected between December 2009 and November 2013 from patients undergoing radical or partial nephrectomy for ccRCC (in collaboration with the Department of Urology and Urological Oncology, Poznan University of Medical Sciences). The study was approved by Local Bioethical Committee at Poznan University of Medical Sciences (no. 1124/12) and all participants gave written informed consent. Samples were stored at −80°C.

The samples used in this study originated from 83 histopathologically confirmed sporadic ccRCC tumors from patients ranging from 30-87y of age, with a median of 71y, at diagnosis while mean follow-up time was 33 months. Disease progression was characterized as local neoplasm recurrence or distant metastasis. Pathological and clinical data are presented in Table 4 and Supplementary Table 5. Control kidney tissue was collected from areas histopathologically identified as non-tumor tissue, from 12 randomly selected patients.

### Expression studies

Real-time PCR-based analysis of *VHL*, *HIF1A* and *EPAS1* genes expression was performed using SYBR® Green master mix (LifeTechnologies) and the Eco Real-Time PCR System (Illumina Inc., San Diego, CA).

### SNP array assay

83 tumor samples and 12 non-tumor controls were analyzed using HumanOmniExpress12v1.1 SNP array (733K BeadChip of Illumina Inc., San Diego, CA). Genomic DNA was isolated from homogenized tissue using Gene MATRIX Universal DNA Purification Kit (EurX, Gdańsk, Poland) following manufacturer's protocol. 200 ng of DNA was used as an input for a single array. DNA amplification, tagging and hybridization were performed according to the manufacturer's protocol. The array slides were scanned on a HiScan SQ (Illumina Inc., San Diego, CA).

Data analysis was performed using GenomeStudio version 2011.1 and Nexus Copy Number 7.0-7.5 (BioDiscovery, El Segundo, CA, USA) with SNP-FASST2 Segmentation. The HapMap control set provided by the manufacturer was used as a control. Standard settings for SNP arrays in Nexus were adjusted: homozygous frequency threshold of 0.95 and minimum loss of heterozygosity (region with LOH) length of 2000 kb and >50 probes were set. QC measurement in Nexus was used as a measure for the array profile quality. Samples with QC < 0.13 were further analyzed. All samples met quality criteria for further analysis. The resolution of 500 kb was generally applied. UCSC built Hg19 (Human Mar. 2009 (NCBI37/hg19) Assembly) was used to analyze the data. The SNP array data of our project is deposited in Nexus DB Biodiscovery (http://www.biodiscovery.com/genomic-databases-nexus-db) under project name: ccRCC_Kluzek_[publication date].

### Analysis of chromosomal aberrations in circulating cell free DNA (cfDNA)

Circulating cell free DNA (cfDNA) from serum samples of 4 patients with ccRCC, 7 patients without chromosomal aberrations (reference set) and a control sample (trisomy 21) was extracted with QIAamp® Circulating Nucleic Acid Kit (QIAGEN) according to the manufacturer's instructions. Concentration of cfDNA was measured on Qubit® fluorometer using the dsDNA HS Assay Kit (Life Technologies, Carlsbad, CA, USA). Libraries were constructed using TruSeq® DNA Library Sample Preparation Kit (Illumina Inc., San Diego, CA) and/or NEBNext® Ultra™ DNA Library Prep Kit for Illumina (New England BioLabs Inc.). Libraries were quantified on Qubit and the quality was assessed using a bioanalyzer on a DNA 1000 Chip (Agilent Technologies). The libraries in 15 pmol concentration were sequenced on the HiScanSQ platform (Illumina, Inc., San Diego, CA), with Reagent Kits v2, 1×50 bp reads. The sequenced reads were mapped to the reference genome NCBI37/hg19 using BWA with zero mismatches allowed, duplicates were removed with SAMtools and resulting BAM files were analyzed with WISECONDOR [46].

### *PBRM1*, *BAP1* and *KDM5C* amplicon sequencing in tumor samples

28 individual primer pairs (Supplementary Table 6) designed in PrimerBlast or OptimusPrimer (Pharmacogenomics Centre; http://op.pgx.ca) software were used to amplify the complete coding sequences and adjacent exon–intron boundaries of the *PBRM1*, *BAP1* and *KDM5C* genes (Supplementary Figure 1) in 95 samples (83 tumors and 12 controls). PCR amplification was performed using KAPA HiFi polymerase (Kapa Biosystems) in a 20μL reaction mix with 50ng of genomic DNA. All amplicons ranging from 1599 to 5341 bp in length were amplified using DNA Engine Dyad^®^ Peltier Thermal Cycle (BioRad). All PCR products were examined by electrophoresis in ethidium bromide stained 0.7% agarose gel.

2nM of amplicons from each of the 28 PCR reactions were pooled and purified with AmpureXP beads (Beckman Coulter, Krefeld). DNA concentration of pooled samples was determined using the HS dsDNA Assay Kit (Invitrogen, Paisley, UK) and measured with a Qubit 2.0 Fluorometer (Invitrogen). NexteraXT DNA Sample Prep Kit (Illumina Inc., San Diego, CA) was used for library preparation, following standard protocol. Libraries were pooled at equimolar concentration and sequenced on an Illumina MiSeq (PE, v2, 2×75 reads) with a 15% PhiX spike‐in.

FASTQ files were subjected to error correction using Blue with default parameters [55]. K-mer length was set to 25. Flexbar was used to remove adapters, low quality reads (Phred <30), over-represented sequences, trim read ends (15 bases at the 5′ end, 10 bases trimmed at the 3′ end), and to remove short sequences (less than 105 bp) [56]. Mapping of purified reads to the human reference genome (NCBI37/hg19 Assembly) was performed for each sample separately using Soap3-dp aligner with default parameters [57].

StrandNGS (Strand Life Sciences) software was used for variant calling, with default analysis settings and dbSNP human Build 146, COSMIC and ClinVar databases were used for identification of known variants. Calls were then filtered based on a score specified in StrandNGS (range 0-1000). Variants with score ≥800 were used for subsequent analyses, after confirmation by direct visual inspection of amplicon reads in Integrative Genomics Viewer. Due to difficulties in accurate mapping of short reads containing complex variations, especially insertions and deletions (indels), DNA variants encompassing >3bp were validated using Sanger sequencing [58]. Non-synonymous variants within coding sequence were further analyzed for the likelihood of functional impact. Predictions were performed with two different tools: MutationAssessor (http://mutationassessor.org/r3/) and Variant Effect Predictor (VEP) from Ensembl database (http://www.ensembl.org).

### Statistical analysis

Association of the chromosomal aberrations with metastasis, disease stage, tumor size or survival was analyzed using Fisher's exact test, Kaplan–Meier survival analysis and the log rank test (GraphPad Prism Software 5.0, San Diego, CA). Statistical tests were two sided with default significance level of 0.05. Univariate logistic regression and univariate Cox regression were performed using R statistical software v3.2.0 as described previously [59].

## SUPPLEMENTARY MATERIALS FIGURES AND TABLES












