# Supplementary material for: Genetic characterization of Polish ccRCC patients: somatic mutation analysis of PBRM1, BAP1 and KDMC5, genomic SNP array analysis in tumor biopsy and preliminary results of chromosome aberrations analysis in plasma cell free DNA
Source: Oncotarget. 2017 Feb 15;8(17):28558–74. doi: 10.18632/oncotarget.15331 (PMC5438672; doi:10.18632/oncotarget.15331)
Supplement: Supplementary file 1 [file oncotarget-08-28558-s001.pdf]

Genetic characterization of Polish ccRCC patients: somatic mutation analysis of *PBRM1*, *BAP1* and *KDMC5*, genomic SNP array analysis in tumor biopsy and preliminary results of chromosome aberrations analysis in plasma cell free DNA

SUPPLEMENTARY FIGURE AND TABLES

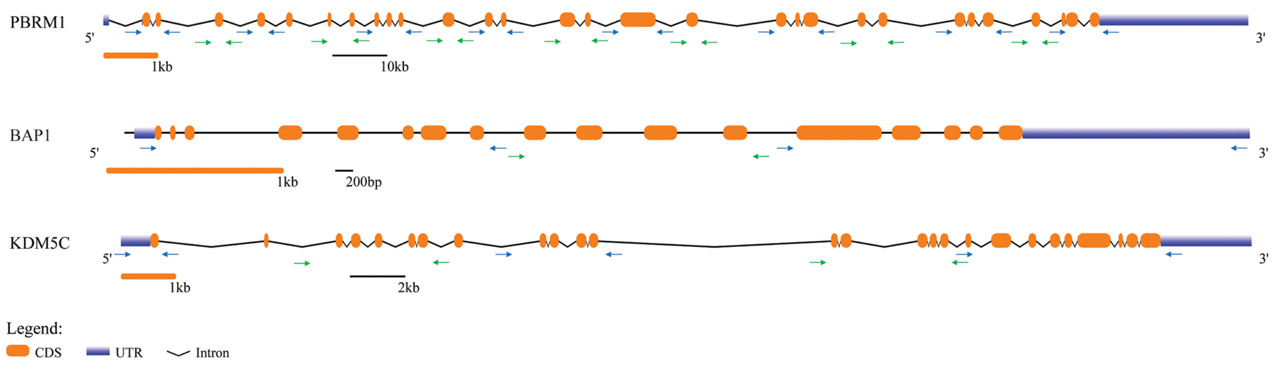

Supplementary Figure 1: Primer location within gene sequence.

Supplementary Table 1: Correlation of chromosomal aberrations frequency with Fuhrman grade. Numbers in parentheses indicate number of patients and male/female ratio, respectively

Aberration type	Fuhrman1 (n=6) (m/f=2/4)	Fuhrman2 (n=26) (m/f=14/12)	Fuhrman3 (n=29) (m/f=11/18)	Fuhrman4 (n=16) (m/f=9/7)
1p deletion (including RUNX3 and ARID1A)	16.7% (1)	3.8% (1)	10.3% (3)	25% (4)
1q gain	0.0 % (0)	11.5% (3)	17.2% (5)	12.5% (2)
2q gain (including ZNF804A)	16.7 % (1)	7.7% (2)	6.9% (2)	0% (0)
2q deletion	16.7 % (1)	7.7% (2)	10.3% (3)	18.75% (3)
3p deletion (including VHL)	100.0 % (6)	76.9% (20)	89.7% (26)	68.75% (12)
3q gain	0.0 % (0)	0.0% (0)	13.8% (4)	6.25% (1)
4q deletion (including NEIL2) or monosomy 4	33.3 % (2)	19.2% (5)	34.5% (10)	25% (4)
5p gain (5p15)	16.7 % (1)	26.9% (7)	13.8% (4)	12.5% (2)
5q gain (including CSF1R)	33.3 % (2)	46.2% (12)	48.3% (14)	12.5% (2)
5q gain (including STC2)	33.3 % (2)	53.8% (14)	51.7% (15)	18.75% (3)
5q gain (including VCAN)	0.0 % (0)	23.1% (6)	17.2% (5)	6.25% (1)
6q deletion (including PARK2) or monosomy 6	0.0 % (0)	26.9% (7)	31.0% (9)	18.75% (3)
7q gain (including NAMPT and MCM7) or trisomy 7	16.7 % (1)	19.2% (5)	41.4% (12)	18.75% (3)
8p deletion (including DLC1 and NRG1)	33.3 % (2)	19.2% (5)	34.5% (10)	31.25% (5)
8p deletion (including DLC1, NRG1 and SFRP1)	0.0 % (0)	19.2% (5)	10.3% (3)	18.75% (3)
8q gain (including COL14A1)	0.0 % (0)	0.0% (0)	17.2% (5)	6.25% (1)
9p deletion (including CDKN2A) or monosomy 9	0.0 % (0)	15.4% (4)	37.9% (11)	31.25% (5)
10q deletion (including KLLN)	16.7 % (1)	3.8% (1)	6.9% (2)	12.5% (2)
12q gain or trisomy 12 (including CDK4 and NDUFA4L2)	0.0 % (0)	11.5% (3)	13.8% (4)	6.25% (1)
13q gain (including EDNRB)	16.7 % (1)	3.8% (1)	6.9% (2)	0% (0)
13q deletion (including RB1)	0.0 % (0)	3.8% (1)	10.3% (3)	25% (4)
Monosomy 14	0.0 % (0)	30.8% (8)	48.3% (14)	37.5% (6)
16q gain (including CDH1)	16.7 % (1)	11.5% (3)	3.4% (1)	6.25% (1)
17p deletion (including BP53)	0.0 % (0)	0.0% (0)	10.3% (3)	18.75% (3)
18q deletion (including DCC)	0.0 % (0)	3.8% (1)	24.1% (7)	25% (4)
20q gain (including E2F1 and HCK) or trisomy 20	0.0 % (0)	7.7% (2)	13.8% (4)	12.5% (2)
Monosomy 22	0.0 % (0)	7.7% (2)	6.9% (2)	25% (4)
Y chromosome loss (in 17 out of 45 male patients)	50.0 % (1)	50.0% (7)	44.4% (8)	10% (1)
Chromothripsis (>4 breakpoint on one chromosome arm)	0.0% (0)	19.2% (5)	37.9% (11)	18.75% (3)

M - male, F - female, NA - not available.

Supplementary Table 2: Common variants detected in *KDM5C*, *BAP1* and *PBRM1* genes. Gray shaded area indicates samples with novel types of variants, but overlapping in terms of chromosomal position with SNPs present in dbSNP.

See Supplementary File 1

Supplementary Table 3: Rare variants detected in *KDM5C*, *BAP1* and *PBRM1* genes.

See Supplementary File 2

Supplementary Table 4: Variant effects analysis using MutationAssesor (MA, light grey shaded area) and Variant Effect Predictor (VEP, green shaded area). Correlation with clinicopathological data and *VHL* status is also shown.

See Supplementary File 3

Supplementary Table 5: Clinical and pathological data of patients with ccRCC (n=83).

See Supplementary File 4

Supplementary Table 6: Primers sequences.

See Supplementary File 5
